# Severe combined immunodeficiency: case report of alogenic, haploidentical bone marrow transplantation

**DOI:** 10.1186/1939-4551-8-S1-A122

**Published:** 2015-04-08

**Authors:** Fernanda Silveira, Liziane Nunes, Paula Lauria, Abelardo Neto, Sandra Bastos, Flavia Anisio, Patricia Lima, Franciane Silva, Juliana Fernandes, Celso Ungier

**Affiliations:** 1 Instituto Nacional De Saude Da Mulher, Da Criança e Do Adolescente Fernandes Figueira - IFF/ Fiocruz, Brazil; 2 Hospital Israelita Albert Einstein, Brazil

## Background

Severe combined immunodeficiency (SCID) is characterized by important impairment in differentiation of T and/or B lymphocytes and occasionally Natural Killer cells, representing a pediatric emergency. A case of immunodeficiency is described emphasizing symptoms, diagnosis and answer after bone marrow transplantation.

## Methods

Case report of a 2 years old male patient with severe combined immunodeficiency (SCID), diagnosed at 9 months after hospitalization due to failure to thrive, chronic diarrhea and pneumonia. Evolved with recurrent respiratory and gastrointestinal infections although using prophylaxis and immunoglobulin infusion. Alogenic, haploidentical transplantation was carried out with positive selection of CD34+, at 18 months of age due no compatible donor been found.

## Results

Satisfactory answer after transplantation keeping infusion of IV immunoglobulin with clinical and laboratorial favorable evolution.

## Conclusions

Bone marrow transplantation when succeeds is supposed to restore lymphocyte system diminishing risks of severe and fatal infections.

## Consent

Written informed consent was obtained from the patient for publication of this abstract and any accompanying images. A copy of the written consent is available for review by the Editor of this journal.

